# Recommendations for open data science

**DOI:** 10.1186/s13742-016-0127-4

**Published:** 2016-05-18

**Authors:** Melissa Gymrek, Yossi Farjoun

**Affiliations:** Program in Medical and Population Genetics, Broad Institute of MIT and Harvard, Cambridge, MA USA; Analytic and Translational Genetics Unit, Massachusetts General Hospital, Boston, MA USA; Data Science and Data Engineering, Broad Institute of MIT and Harvard, Cambridge, MA USA

**Keywords:** Reproducible research, Open science, Best practices

## Abstract

Life science research increasingly relies on large-scale computational analyses. However, the code and data used for these analyses are often lacking in publications. To maximize scientific impact, reproducibility, and reuse, it is crucial that these resources are made publicly available and are fully transparent. We provide recommendations for improving the openness of data-driven studies in life sciences.

## Background

The ability to generate increasingly large and complex datasets has ushered in an era of ‘big data’ in life sciences and in other fields such as astronomy, economics, and physics. The field of data science has emerged to tackle the challenge of extracting information from these data sets by combining aspects of statistics, machine learning, software engineering, and data visualization. Data science has been accompanied by a number of ‘open’ movements aimed at maximizing scientific impact by increasing accessibility of science, promoting reproducibility and enabling future studies. These include the sharing of raw data sets (open data), source code (open source), publications (open access), and education (open teaching). The life sciences community has long recognized the importance of open data [1], and has recently reinforced its importance [2]. Methods used to generate results are equally important, but availability of tools and workflows are often not required for publication. For scientific progress, it is critical that the life sciences undergo a cultural shift whereby computational methods are held to similarly high standards.

We have provided recommendations for open data science to facilitate this shift.

## Recommendations

### 1. Provide or cite source code for tools in public repositories

Software packages are critical in data-driven studies. Other researchers can only fully utilize results if the details of the computational methods used are completely transparent. Provide source code for all original software in public repositories such as Github [3] with a license permitting as free use as possible. Cite precise versions and command line prompts for all previously published tools. Do not use proprietary software that cannot be accessed by other researchers.

Follow best practices in software engineering [4] to make tools easy to use and maintain by others. A common hurdle for reusing software is the cumbersome and heterogeneous steps required to install a specific program. To alleviate this issue, deposit tools in standard package managers, such as the Python Package Index (PyPI) for Python or the Comprehensive R Archive Network (CRAN) for R. Alternatively, provide instructions for easy installation using standard tools.

Taking these steps to ensure that software is publicly available and user-friendly will ultimately result in much greater impact by making the software more widely used, and thus more likely to be improved and maintained by the community.

### 2. Provide or cite pipelines in public repositories

Workflows integrating diverse data sources and individual tools to generate numerical results, figures, or tables, are important components of many computational analyses. However, although publications often describe these workflows in varying levels of detail, the pipelines implementing them are rarely shared, despite being fundamental for understanding how results were generated and for repeating analyses.

As ‘big data’ analyses become more widespread, best practices for publishing pipelines are still evolving. Full transparency requires that all code, data, and steps be shared in formats that allow another researcher to easily regenerate the same results. For analysis of large data sets using standard tools, use citable pipelines (e.g. Galaxy [5]), or a standardized pipeline language such as Yet Another Workflow Language (YAWL) [6]. For smaller pipelines, post documented source code at a stable website, such as a Git repository, containing scripts and links to data that is specific to a manuscript. This code could take the form of reproducible notebook tools such as Jupyter notebooks [7] or Sweave/knitr [8] scripts, which integrate code with results and figures. Another option is to provide a script or makefile that can regenerate all analyses of a paper with a single command. Finally, containerizing analyses (e.g. using Docker [9]) can allow them to be easily rerun by others without having to install additional dependencies.

### 3. Train scientists in data science

A critical element of a cultural revolution toward high standards for data analysis will be engaging young scientists in data science training. Valuable new tools are emerging for facilitating open data science such as Github [3] for sharing code, and Project Jupyter notebooks [7] for publishing reproducible analyses. However, students rarely receive formal training in current data science tools and techniques. Moreover, they often face pressure to produce results as quickly as possible. As a result, scientists may produce hastily and poorly written code that they are reluctant to share and is unusable by others.

The primary source of training often comes from the laboratory environment itself. For wet-lab work, students are usually trained in practices such as keeping a lab notebook and sharing protocols. Similar efforts must now be invested for computational work. For instance, tools and analyses should be reviewed within the lab for quality and correctness. One suggestion is to maintain a lab Github repository where analyses are discussed as ‘issues’ and peer-reviewed via ‘pull-requests’. Another suggestion is to hold lab-wide or institute-wide data science tutorials. Finally, trainees can take advantage of online learning platforms (e.g. Software Carpentry [10]) that provide freely available training in tools and practices for data-driven research.

### 4. Journal editors and reviewers must enforce computational reproducibility

These recommendations will likely only take effect if required for publication. Many journals encourage authors to publish source code and data, but often do not have specific requirements about what should be provided, and how. For instance, *Nature* instructs authors to include a ‘Code Availability’ section in their manuscripts to indicate ‘whether and how the code can be accessed’. *PLoS* posts guidelines for requiring source code, documentation, and test data sets, although these refer specifically to manuscripts describing new software. *GigaScience* goes a step further with more specific requirements for code, data, and workflows, and states that ease of reproducibility is a key criterion during the review process. Importantly, editors must enforce these guidelines by following through with authors’ promises to post code and data.

Reviewers also have a responsibility to ensure that computational methods are adequately reported. One suggestion is that journals provide authors and reviewers with a ‘computational reproducibility checklist’, similar to checklists already provided for statistical analyses. This checklist may ask whether the source code and data for all tools and analyses are available, whether new tools have been deposited in public package managers, and whether sufficient documentation and user manuals are available. Failure to comply with this checklist should disqualify a study from publication. Requiring more thorough review of code and analyses takes additional time that most reviewers are unwilling to spend, especially if new tools are difficult to install and don’t work ‘out of the box’. A suggestion to reduce this burden is to designate specific data science reviewers. Requiring specific review of these aspects will incentivize scientists to invest greater effort in the practices outlined above.

## Conclusions

Making computational research fully reproducible, transparent, and reusable is challenging, time consuming, and is often a low priority for researchers. Nevertheless, investing efforts in establishing best practices and standards for sharing computational analyses will ultimately move science forward faster.

This revolution will not occur by itself. It will require a concerted effort by trainees, senior scientists, editors, and reviewers, as was the case for previous revolutions in data sharing and statistics. These recommendations offer a potential springboard for the policies and mental shift needed across the field to make the ideals of open data science a reality.

## Abbreviations

CRAN: comprehensive R archive network
PyPI: python package index
YAWL: yet another workflow language
